# Supplementary material for: A Regulatory Noncoding RNA, nc886, Suppresses Esophageal Cancer by Inhibiting the AKT Pathway and Cell Cycle Progression
Source: Cells. 2020 Mar 26;9(4):801. doi: 10.3390/cells9040801 (PMC7226379; doi:10.3390/cells9040801)
Supplement: Supplementary file 1 [file cells-09-00801-s001.zip › Manuscript ID cells_689847_Supple_Cells_rev_final.DOCX]

**Supplementary Information (Figure S1-S2)**

**(Im, et al: “A regulatory noncoding RNA, nc886, suppresses esophageal cancer by inhibiting the AKT pathway and cell cycle progression”)**


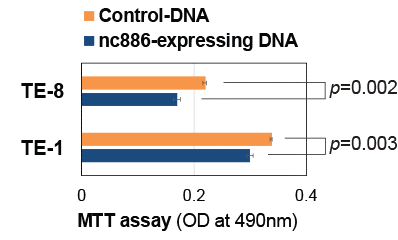


**Figure S1. nc886 inhibits ESCC cell proliferation.**

MTT assays at 24 hrs after transfection of “nc886-expressing DNA” and “Control-DNA”. nc886-expressing DNA is a PCR product of 667-nucleotides (nts) harboring the nc886 and flanking sequences. Control-DNA is also a PCR product with irrelevant sequence. Sequences of PCR primers are (5' to 3'): “ggggtcgacctgctggacctaggtagacg” and “gggactagtaatccataacgcactccgcg” (for nc886-expressing DNA); “gggggggatccgattgctatcagcaatgccccaaac” and “ggggggaattcatcgctgtgcagtgttctcccaga” (for Control-DNA). 200 ng of each DNA per a 96-well was transfected into indicated cells by Lipofectamine™ 2000 reagent (Invitrogen; Carlsbad, CA). An average and the standard error were calculated from triplicate measurement. p-values from Student's T-test are also shown.

In two ESCC cell lines TE-1 and TE-8, transfection of nc886-expressing DNA resulted in decrease of cell proliferation. This result corroborates our previous report [[1](#_ENREF_1)], in which ESCC cell proliferation was decreased also by transfection of nc886 RNA. The adverse effect of nc886 on cell proliferation indicates that nc886 plays a tumor suppressor role and explains why we failed to construct an ESCC cell line ectopically expressing nc886.


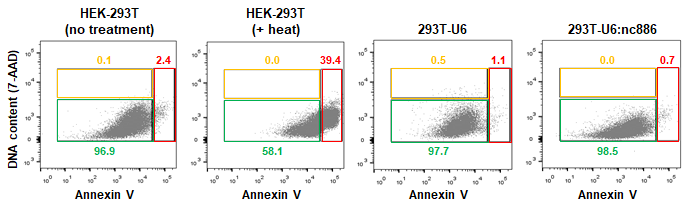
**Figure S2. nc886 does not induce apoptosis**

Apoptotic or necrotic cells were discriminated from live cells by FACS analysis, after cells were stained with Annexin V and 7-AAD using APC Annexin V Apoptosis Detection Kit with 7-AAD (BioLegend; San Diego, CA) according to the manufacturer’s instructions. Each zone of live, apoptotic and necrotic cells is indicated by green, red, and yellow boxes respectively. To ensure whether the apoptotic zone was correct, apoptosis was artificially induced by heating cells at 55°C for 20 min (the second box indicated by “heat”). Apoptotic cells were barely (0.7%) detectable in growing 293T-U6:nc886 cells. 0.7% is no higher than 1.1 % of 293T-U6 cells.

**A**

**
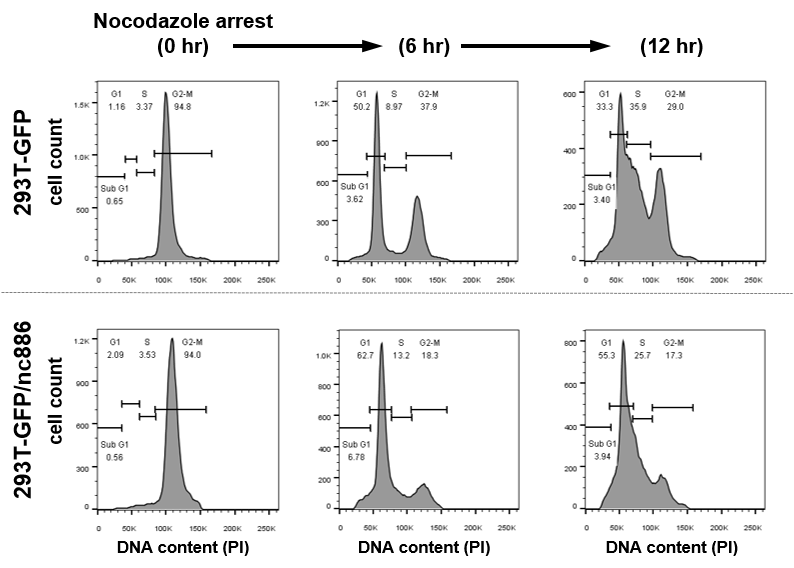
**

**B**


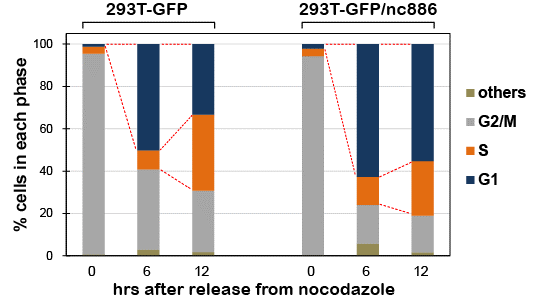


**Figure S3. nc886 delays G1-S transition**

**A-B**. FACS cell cycle profiles were measured as described in the main text (see Materials and Methods, Results, and Fig 2 legend). The difference from Fig 2 is the used cell line pair; 293T-GFP and 293T-GFP/nc886 (this figure) versus 293T-U6 and 293T-U6:nc886 (**Fig 2**). Cells were nocodazole-arrested at G2/M phase and released for entering to G1 and on. Cells were harvested for propidium iodide (PI) staining of DNA contents, just prior to release (0 hr) and 6 and 12 hrs afterwards (panel A). From the FACS profile, % of cells in each phase were counted and plotted (panel B).

In both cell lines, the majority of cells entered into G1 at 6 hr. At 12 hr, the S phase population was higher in 293T-GFP than in 293T-GFP/nc886. This result is consistent with Fig 2 data and reassures that G1-to-S transition becomes slow in nc886-expressing cells.


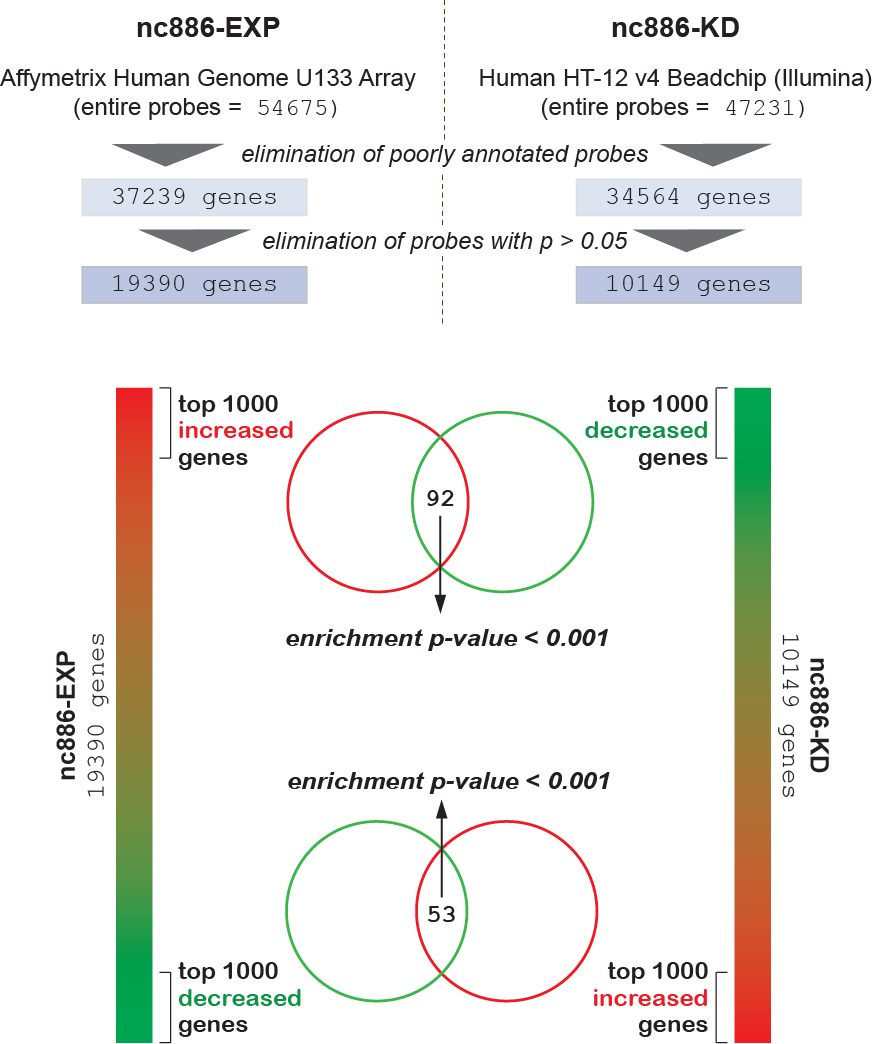


**Figure S4. The top altered genes from nc886-EXP and nc886-KD overlap significantly.**

The workflow (on the top) depicts array data processing for comparison of genes and calculation of enrichment p-values (on the bottom). From raw array data, we made a short-list of genuine genes by eliminating poorly annotated genes such as EST clones or hypothetical proteins (mostly designated with a KIAA-, FLJ-, DKFZ-, LOC-, or MGC-clone number). This processing yielded 37,239 and 34,564 genes for nc886-EXP and nc886-KD respectively. Among them, 19,390 and 10,409 genes (52% and 29% for nc886-EXP and nc886-KD) were significantly changed (with a p-value cutoff = 0.05). We selected 1,000 top altered genes from each dataset and compared them reciprocally. The Venn diagrams show significant overlap, with enrichment p-values (calculated by Fisher's exact test) < 0.001.

**
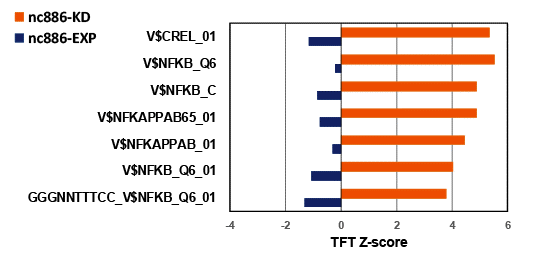
**

**A**

**
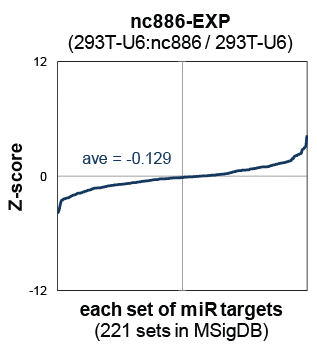
**

**B**

**Figure S5. nc886-EXP barely affects NF-κB target gene sets and the microRNA (miRNA) pathway**

**A**. A bar graph showing Z-scores for NF-κB in TFT gene sets. They are significantly enriched in nc886-KD but not in nc886-EXP.

**B**. In our recent study [[2](#_ENREF_2)], nc886 leads to global suppression of the miRNA pathway in ovarian cancer cells, by inhibiting Dicer that is a key enzyme in the miRNA biogenesis. We interrogated whether nc886 exerts the same function in 293T cells. In the Molecular Signature Database (MSigDB: <http://software.broadinstitute.org/gsea/msigdb>), there are a total of 221 MIR sets each of which is a collection of genes that have a miRNA target seed sequence(s) in their 3'-untranslated region. If nc886 inhibited Dicer and therefore down-regulated miRNAs in 293T cells, MIR-Z scores of nc886-EXP (293T-U6:nc886 values relative to 293T-U6) would shift towards positive values, because a miRNA suppresses the expression of its target mRNAs (whose collection is a MIR set).

The graph is a rank distribution plot of MIRs (miRNA target gene sets). All MIR Z-scores in nc886-EXP were sorted in an ascending order and were plotted against an anonymous x-axis, to visualize their overall distribution. The plot shows that MIRs in nc886-EXP did not tend to have positive values. The number of MIRs of positive Z-scores was nearly identical to those with negative values. In addition, only few MIRs were changed significantly (Z-score >+3 or <-3; see Table S3). Therefore, the miRNA pathway appeared to be barely affected by nc886 in 293T cells, unlike ovarian cancer cells.


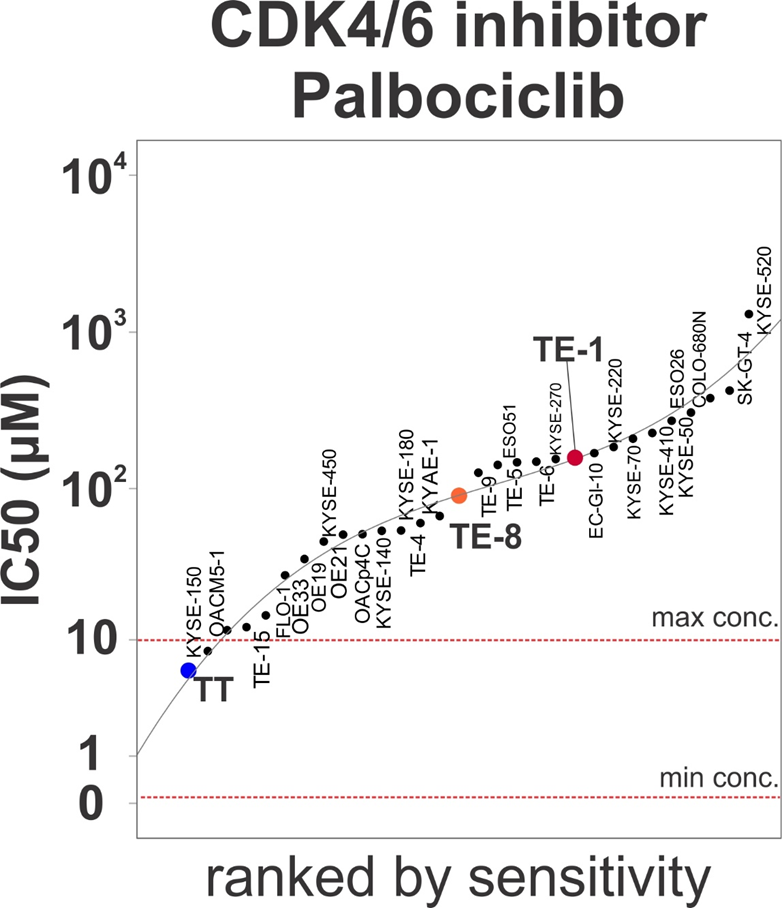


**Figure S6. Palbociclib sensitivity from a public database**

A rank plot of IC50 values of 30 esophageal cancer cell lines. We examined the sensitivity to palbociclib, an FDA-approved CDK4/6 inhibitor, in the Genomics of Drug Sensitivity in Cancer Database ([www.cancerRxgene.org](http://www.cancerRxgene.org)), the largest public resource for information on the sensitivity of almost 1,000 cancer cell lines to 100s of compounds [[3](#_ENREF_3),[4](#_ENREF_4)]. The order of IC50 values, which are TE-1 > TE-8 > TT, was the same as our experimental data (**Fig 7D**).

**Supplemental References**

1. Lee, H.S.; Lee, K.; Jang, H.J.; Lee, G.K.; Park, J.L.; Kim, S.Y.; Kim, S.B.; Johnson, B.H.; Zo, J.I.; Lee, J.S., et al. Epigenetic silencing of the non-coding RNA nc886 provokes oncogenes during human esophageal tumorigenesis. *Oncotarget* **2014**, *5*, 3472-3481, doi:10.18632/oncotarget.1927.

2. Ahn, J.H.; Lee, H.S.; Lee, J.S.; Lee, Y.S.; Park, J.L.; Kim, S.Y.; Hwang, J.A.; Kunkeaw, N.; Jung, S.Y.; Kim, T.J., et al. nc886 is induced by TGF-beta and suppresses the microRNA pathway in ovarian cancer. *Nature communications* **2018**, *9*, 1166, doi:10.1038/s41467-018-03556-7.

3. Yang, W.; Soares, J.; Greninger, P.; Edelman, E.J.; Lightfoot, H.; Forbes, S.; Bindal, N.; Beare, D.; Smith, J.A.; Thompson, I.R., et al. Genomics of Drug Sensitivity in Cancer (GDSC): a resource for therapeutic biomarker discovery in cancer cells. *Nucleic Acids Res.* **2013**, *41*, D955-961, doi:10.1093/nar/gks1111.

4. Garnett, M.J.; Edelman, E.J.; Heidorn, S.J.; Greenman, C.D.; Dastur, A.; Lau, K.W.; Greninger, P.; Thompson, I.R.; Luo, X.; Soares, J., et al. Systematic identification of genomic markers of drug sensitivity in cancer cells. *Nature* **2012**, *483*, 570-575, doi:10.1038/nature11005.
